# Movement velocity can be used to estimate the relative load during the bench press and leg press exercises in older women

**DOI:** 10.7717/peerj.7533

**Published:** 2019-08-20

**Authors:** Pablo Jorge Marcos-Pardo, Jorge Miguel González-Hernández, Amador García-Ramos, Abraham López-Vivancos, Pedro Jiménez-Reyes

**Affiliations:** 1 Faculty of Sport, Catholic University San Antonio of Murcia, Murcia, Spain; 2 Faculty of Health Science, European University of Canarias, Tenerife, Islas Canarias, Spain; 3 Faculty of Sport Sciences, University of Granada, Granada, Spain; 4 Department of Sports Sciences and Physical Conditioning, Faculty of Education, CIEDE, Universidad Católica de la Santísima Concepción, Concepción, Chile; 5 Centre for Sports Studies, Rey Juan Carlos University, Alcorcón, Madrid, Spain

**Keywords:** Linear position transducer, Load-velocity profile, Resistance training, Velocity-based training, Older women

## Abstract

**Background:**

Movement velocity has been proposed as an effective tool to prescribe the load during resistance training in young healthy adults. This study aimed to elucidate whether movement velocity could also be used to estimate the relative load (i.e., % of the one-repetition maximum (1RM)) in older women.

**Methods:**

A total of 22 older women (age = 68.2 ± 3.6 years, bench press 1RM = 22.3 ± 4.7 kg, leg press 1RM = 114.6 ± 15.9 kg) performed an incremental loading test during the free-weight bench press and the leg press exercises on two separate sessions. The mean velocity (MV) was collected with a linear position transducer.

**Results:**

A strong linear relationship between MV and the relative load was observed for the bench press (%1RM = −130.4 MV + 119.3; *r*^2^ = 0.827, standard error of the estimate (SEE) = 6.10%1RM, *p* < 0.001) and leg press exercises (%1RM = −158.3 MV + 131.4; *r*^2^ = 0.913, SEE = 5.63%1RM, *p* < 0.001). No significant differences were observed between the bench press and leg press exercises for the MV attained against light-medium relative loads (≤70%1RM), while the MV associated with heavy loads (≥80%1RM) was significantly higher for the leg press.

**Conclusions:**

These results suggest that the monitoring of MV could be useful to prescribe the loads during resistance training in older women. However, it should be noted that the MV associated with a given %1RM is significantly lower in older women compared to young healthy individuals.

## Introduction

Aging is associated with a progressive decrease of muscle mass and a reduced capacity of the muscles to produce strength and power (Aagaard et al., 2010; Guizelini et al., 2018). The ability of lower extremity muscles to generate force is a fundamental component for maintaining balance (Li et al., 2018). These changes may increase the risk of falls and fractures, which could contribute to the loss of independence and the deterioration in the physical function and quality of life of older adults (Trombetti et al., 2016; Gadelha et al., 2018; Ma et al., 2018). Muscular strength seems to be an extremely important factor associated with the prevention of falls and an increased functional capacity (Balsalobre-Fernández et al., 2018) and is effective for improving physical performance and quality of life of older adults (Ramirez-Campillo et al., 2016). Resistance training is widely recommended to slow down the negative effects of aging because it is considered the most effective method for improving muscular strength and power which are key to counteract frailty in older adults (Sundell, 2010; Cornelissen et al., 2011; Cadore et al., 2014; Chodzko-zajko, 2014). In addition, individuals undertaking resistance training demonstrate an increased motivation and adherence to training (Kekäläinen et al., 2018; Marcos-Pardo, Martínez-Rodríguez & Gil-Arias, 2018).

The one-repetition maximum (1RM) is the main reference to prescribe the load during resistance training (Ratamess et al., 2009). However, the direct determination of the 1RM has been discouraged because it is a time-consuming procedure which is impractical for large groups of subjects, while 1RM value can change quickly as a consequence of training (García-Ramos & Jaric, 2018). For example, old subjects required eight to nine sessions to have consistency in the measurement of the 1RM during the bilateral concentric knee extension exercise (Ploutz-Snyder & Giamis, 2001). The monitoring of movement velocity has been proposed as an alternative to prescribe and quantify the training load during resistance training (Jovanovic & Flanagan, 2014; Mann, Ivey & Sayers, 2015; Balsalobre-Fernández et al., 2018) as well as to increase motivation during training (Wilson et al., 2017; Weakley et al., 2018). One of the main applications of velocity-based resistance training is the use of movement velocity to estimate the 1RM (Banyard, Nosaka & Haff, 2017; García-Ramos et al., 2018a; Perez-Castilla et al., 2019). Previous studies have identified a strong and linear relationship between the relative load (%1RM) and movement velocity in a variety of resistance training exercises such as the bench press, leg press, squat, bench pull, or deadlift (Sánchez-Medina et al., 2014; Conceição et al., 2016; Helms et al., 2017; García-Ramos et al., 2018b, 2019; Pérez-Castilla et al., 2018). However, it has been suggested that the accuracy decreases when the exercises are performed with free-weights instead of using a Smith machine (Banyard, Nosaka & Haff, 2017; Hughes et al., 2018). These results open the possibility of determining the 1RM without the need of performing a maximal lift which is specially discouraged for vulnerable populations such as older adults (Ploutz-Snyder & Giamis, 2001). However, it is important to note that the vast majority of studies have explored the load-velocity relationship in young healthy men and women (Torrejon et al., 2018; Balsalobre-Fernández, García-Ramos & Jiménez-Reyes, 2018; Perez-Castilla et al., 2019), and no study has examined the load-velocity relationship in the older adults.

The force-velocity relationship has been recommended to determine the maximal capacities of the muscles to produce force, velocity and power in older people (Alcazar et al., 2017, 2018). However, it should be noted that the force-velocity and load-velocity relationships do not provide the same information (García-Ramos & Jaric, 2018) and, therefore, it should be elucidated whether the load-velocity relationship can be used to estimate the 1RM and the relative loads (%1RM) in this population. The most similar study that has explored the effect of age on the load-velocity relationship was conducted by Fernandes, Lamb & Twist (2018), who compared the load-velocity relationship during the bench press, squat and bent-over- row exercises between young (age: 21.0 ± 1.6 years) and middle-age men (age: 42.6 ± 6.7 years), and they reported lower velocities for the same %1RM for middle-age men compared to young men. Training at faster velocities with similar relative loads may induce greater improvements in maximal force and greater improvements in older adults ability to improve maximal force and the ability to express higher power outputs and explosive force (da Rosa Orssatto et al., 2018). Therefore, it is plausible that the %1RM-velocity relationship previously determined in young adults would not be applicable to older adults. In this regard, it is important to clarify whether movement velocity could also be an effective tool to prescribe the load (%1RM) in older adults. This would be of great interest for older adults because the direct determination of the 1RM through a single maximal lift is hindered by the high level of expertise required (Gentil et al., 2017).

The purpose of this study was to investigate the relationship between movement velocity and relative load (%1RM) during the bench press and leg press exercises in older women. It was hypothesized that the %1RM would be estimated with an acceptable precision from the recordings of movement velocity during both exercises, while the velocities associated with each %1RM in older women would be lower than the velocities previously reported for young adults. The confirmation of our hypotheses would suggest that the monitoring of movement velocity could be an effective tool to prescribe resistance training in older adults.

## Methods

### Participants

A total of 22 older women (mean ± standard deviation (SD); age = 68.2 ± 3.6 years, body mass = 70.0 ± 12.9 kg, height = 1.6 ± 0.6 m) volunteered to participate in this study. Inclusion criteria were: (a) at least 2 years of resistance training experience, (b) performing resistance training at least two times per week, (c) previous experience with the bench press and leg press exercises, and (d) women between 65 and 75 years of age. Exclusion criteria were: (a) physical limitations or musculoskeletal injuries that could affect tested performance, and (b) taking medications known to influence physical performance. One participant did not attend the leg press testing session and, consequently, the load-velocity relationship was only determined for 21 participants. All participants read and signed a written informed consent to participate in the study that was carried out in accordance with the process approved by the local ethics committee (CE031207) of the Catholic University of Murcia (Spain) and in agreement with the Declaration of Helsinki.

### Study design

A cross-sectional study was designed to determine whether movement velocity is a useful variable for estimating the relative load (%1RM) during the bench press and leg press exercises in older women. The bench press and leg press exercises were chosen because they are two of the exercises most commonly used by older people to increase upper- and lower-body strength, respectively (Fatouros et al., 2005; Bavaresco Gambassi et al., 2019). Following two familiarization sessions with both exercises (participants performed repetitions against different external loads to ensure a proper technique during the experimental sessions), participants came to the laboratory on two more occasions separated by 48–72 h. The bench press and leg press exercises were performed in different sessions in a randomized order. The load-velocity relationship was established on each testing session by means of a standard incremental loading test (Conceição et al., 2016; García-Ramos et al., 2018b). The same experienced researcher supervised all tests and provided real-time velocity feedback to encourage the participants to exert maximum effort. All sessions were performed at the same time of the day for each participant (±1 h) and under similar environmental conditions (~22 °C and ~60% humidity).

### Bench press testing procedure

The testing session began with a standardized warm-up that included 5 min of cycling, upper-body dynamic stretching, and one set of six repetitions against 10 kg (mass of the unloaded barbell used in the testing procedure) during the bench press exercise. The initial external load of the incremental loading test was 10 kg and was progressively increased until the 1RM was achieved. The magnitude of the increment was based on the mean velocity (MV) of the barbell: five kg (MV > 0.80 m · s^−1^), 2.5 kg (0.80 m · s^−1^ ≤ MV ≤ 0.30 m · s^−1^), and one kg (MV < 0.30 m · s^−1^). Participants performed three repetitions with light loads ((MV) > 1.00 m · s^−1^), two repetitions with medium loads (1.00 m · s^−1^ ≥ MV ≤ 0.45 m · s^−1^), and one repetition with heavy loads (MV < 0.45 m · s^−1^). The rest period between successive sets was 5 min. Participants performed an average of 3.8 ± 0.4 sets. The MV of the barbell was measured with a linear position transducer (Chronojump, Barcelona, Spain) that sampled the displacement-time data at a frequency of 1,000 Hz. The cable of the linear position transducer was attached to the right side of the barbell. The highest MV (i.e., average velocity from the start of the concentric phase until the weight reaches the maximum height) of each load was considered for the modeling of the load-velocity relationship. Participants performed the bench press with a free-weight barbell. They were instructed to hold the barbell in contact with their chest parallel to the nipples for 2 s before being instructed to lift the barbell as fast as possible until their elbows reached full extension.

### Leg press testing procedure

The testing session began with a standardized warm-up that included 5 min of cycling, lower-body dynamic stretching, and one set of six repetitions against 30 kg during the leg press exercise. The initial external load of the incremental loading test was 59 kg and was progressively increased until the 1RM was achieved. The magnitude of the increment was based on the MV value: 20 kg (MV > 0.80 m · s^−1^), 10 kg (0.80 m · s^−1^ ≤ MV ≤ 0.30 m · s^−1^), and 2.5 kg (MV < 0.30 m · s^−1^). Participants performed three repetitions with light loads (MV > 1.00 m · s^−1^), two repetitions with medium loads (1.00 m · s^−1^ ≥ MV ≤ 0.45 m · s^−1^), and one repetition with heavy loads (MV < 0.45 m · s^−1^). The rest period between successive sets was 5 min. Participants performed an average of 3.59 ± 0.94 sets. The MV of the barbell was measured with a linear position transducer (Chronojump, Barcelona, Spain) which was attached to the bar on the bench press and to the machine platform to the leg press machine. The highest MV of each load was considered for the modeling of the load-velocity relationship. The concentric phase was performed in a controlled way until they achieved full knee flexion. Then, eccentric phase, explosively as fast as possible when the researcher indicated. A leg press machine (Technogym, Cesena, Italy) with a 45° incline was used.

### Statistical Analyses

Descriptive data are presented as means and SD. The normal distribution of the data was confirmed by the Shapiro–Wilk test (*p* > 0.05). The relationship between MV and %1RM was established by means of linear regression models (Pestaña-Melero et al., 2018). The goodness of fit was assessed by the Pearson's multivariate coefficient of determination (*r*^2^) and the standard error of the estimate (SEE). The MV associated with each %1RM was compared through paired samples *t*-tests and the Hedge's *g* effect size. Finally, the between-subject coefficient of variation (CV) was calculated to determine the variability of the MV associated with each %1RM. Significance was accepted at *p* < 0.05. All statistical analyses were performed using the software package SPSS (IBM SPSS version 22.0; Chicago, IL, USA).

## Results

**Figure 1:**
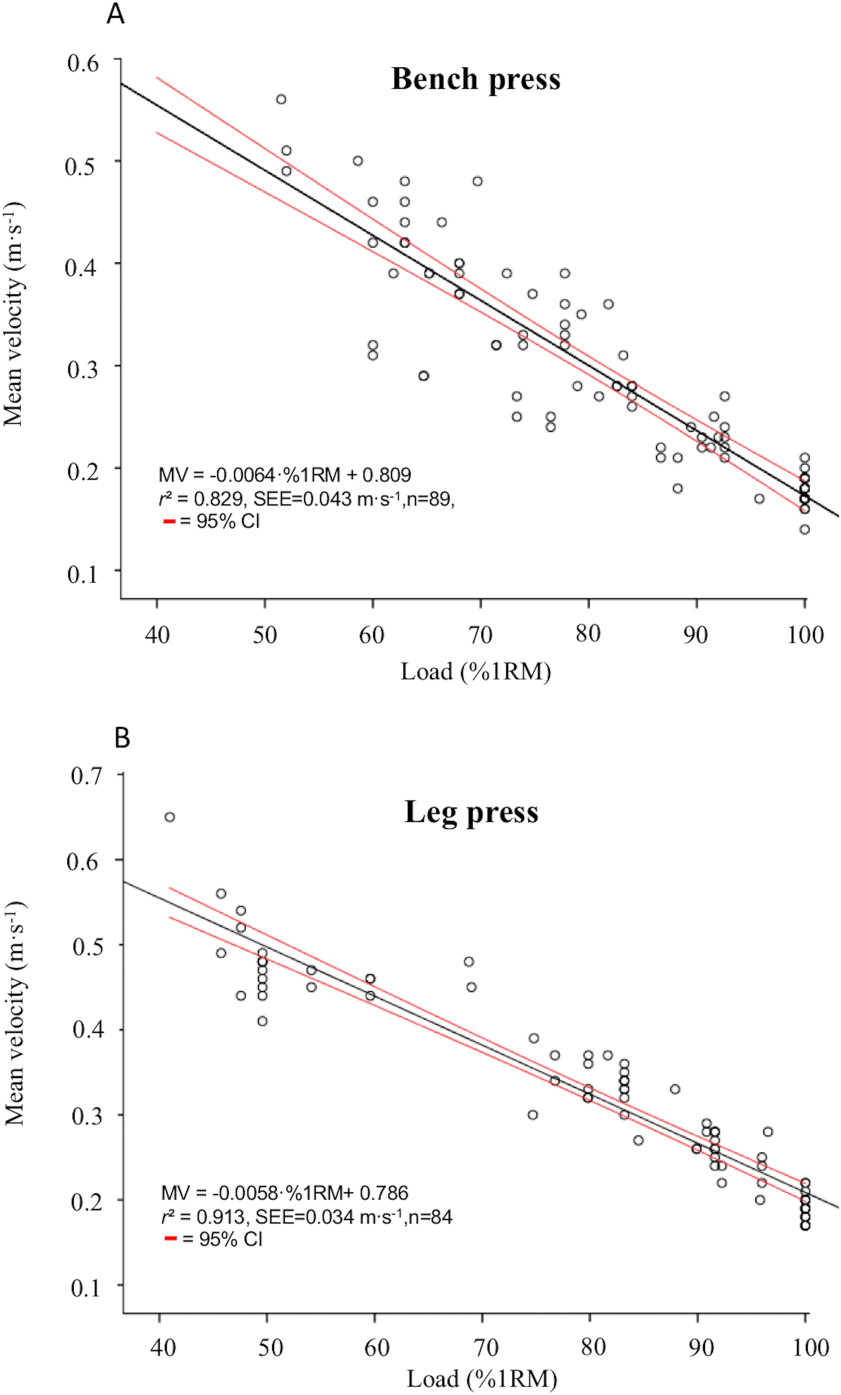
Relationship between relative load (%1RM) and mean velocity (MV) in the bench press (A) and leg press (B). 1RM, one-repetition maximum; *r*^2^, Pearson's multivariate coefficient of determination; SEE, standard error of the estimate; *n* = number of trials included in the regression analysis.

A significant and linear relationship between MV and the relative load (%1RM) was observed in both the bench press and leg press exercises (*p* < 0.001) (Figs. 1A and 1B). Therefore, the following general regression equations could be proposed to estimate the %1RM from MV in older women:

Bench press %1RM = −130.4 × MV + 119.3 (*r*^2^ = 0.827, SEE = 6.10%1RM)
Leg press %1RM = −158.3 × MV + 131.4 (*r*^2^ = 0.913, SEE = 5.63%1RM)

**Figure 2:**
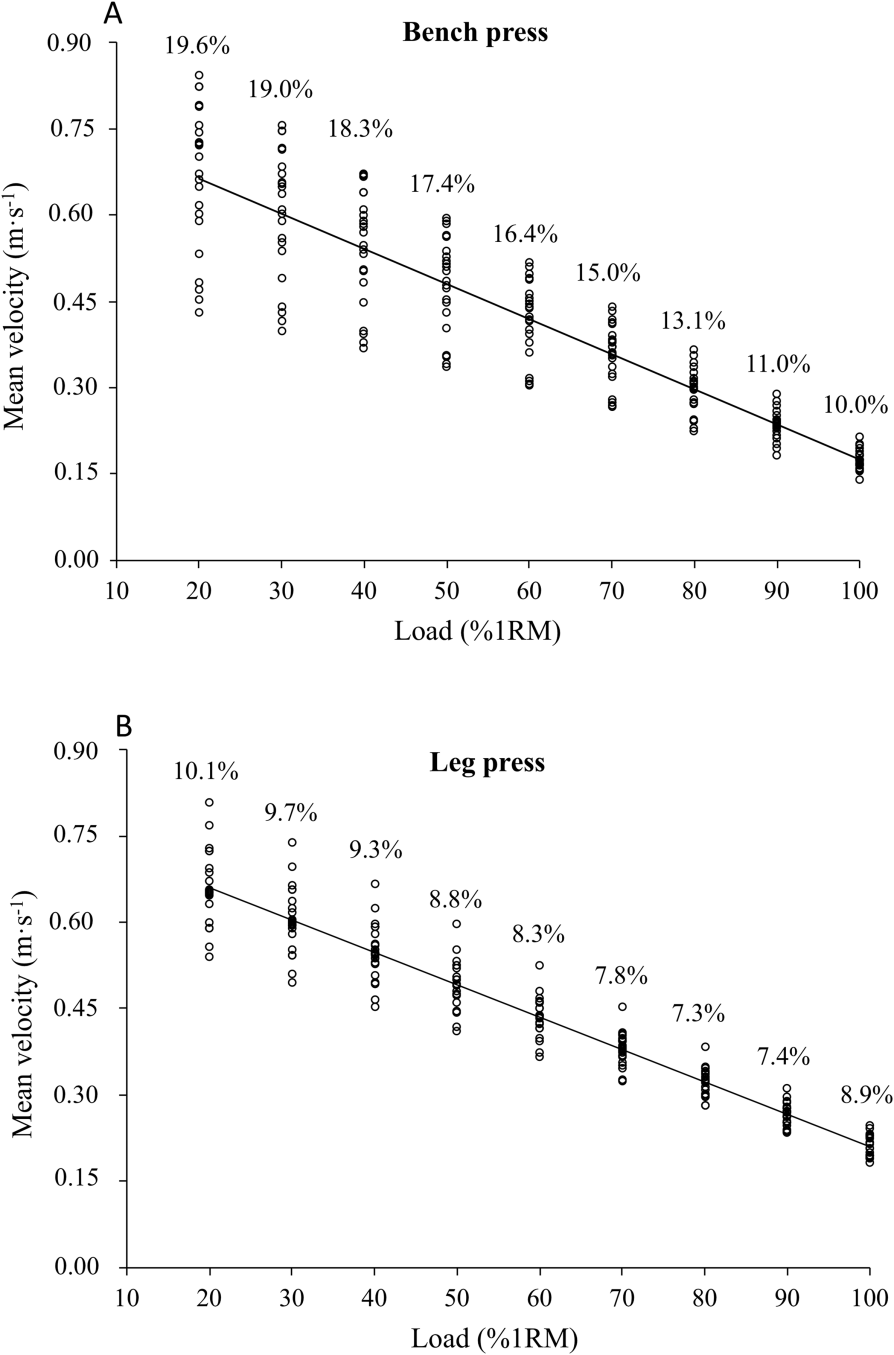
Mean velocities (MV) associated with each relative load (%1RM) obtained from the individual load-velocity relationships in the bench press (A) and leg press (B). Each point represents the data of an individual subject. The numbers depict the between participant coefficient of variation. 1RM, one-repetition maximum.

No significant differences were observed between the bench press and leg press exercises for the MV attained against light-medium relative loads (≤70%1RM), while the MV associated with heavy loads (≥80%1RM) was significantly higher for the leg press (Table 1). Finally, the between-participant variability for the MV associated with each %1RM was generally higher for the bench press (CV = 15.5 ± 3.5%) compared to the leg press (CV = 8.6 ± 1.0%) (Figs. 2A and 2B).

**Table 1:** Comparison of the mean velocity attained against each relative load between the bench press and leg press exercises.

Load (%1RM)	Bench press (m · s^−1^)	Leg press (m · s^−1^)	*p*-value	ES (Hedge's *g*)
20	0.66 ± 0.13	0.66 ± 0.07	0.920	−0.03
30	0.60 ± 0.11	0.60 ± 0.06	0.969	0.01
40	0.54 ± 0.10	0.55 ± 0.05	0.823	0.08
50	0.48 ± 0.08	0.49 ± 0.04	0.631	0.16
60	0.42 ± 0.07	0.44 ± 0.04	0.395	0.28
70	0.36 ± 0.05	0.38 ± 0.03	0.155	0.47
80	0.30 ± 0.04	0.32 ± 0.02	0.019	0.78
90	0.24 ± 0.03	0.27 ± 0.02	<0.001	1.30
100	0.17 ± 0.02	0.21 ± 0.02	<0.001	1.91

**Note:**

1RM, one-repetition maximum; ES, Hedge's *g* effect size (ES = (Leg press mean − Bench press mean)/SD both).

## Discussion

This study was designed to elucidate whether movement velocity could be used to estimate the relative load (%1RM) in older women. Previous studies carried out with young individuals have revealed a nearly perfect relationship between MV and the %1RM during the bench press and leg press exercises (*r*^2^ ≥ 0.94) (Conceição et al., 2016; García-Ramos et al., 2018b, 2019; Loturco et al., 2017; Sánchez-Medina et al., 2014; Torrejon et al., 2018). Based on these results, it is suggested that the %1RM can be predicted with an acceptable level of precision from MV recordings. However, this is the first study that has explored the feasibility of MV to estimate the %1RM in older individuals (>65 years). The main findings of this study revealed that MV can also provide useful information to prescribe the relative load (%1RM) in older women during the bench press and leg press exercises. However, it should be noted that the precision of the load-velocity relationship was weaker (*r*^2^ = 0.83 and SEE = 6.10%1RM in bench press; *r*^2^ = 0.91 and SEE = 5.63%1RM in leg press) compared to what has been reported for young individuals for the same exercises (*r*^2^ ≥ 0.94). In addition, the MV associated with submaximal loads in this study was considerably lower to what has been described for young individuals. These results indicate that although movement velocity can provide useful information for designing resistance training programs in older adults, practitioners should be aware that the MV associated with each %1RM reported in studies conducted with young individuals cannot be extrapolated to older adults.

The bench press has been the most used exercise to explore the load-velocity relationship (García-Ramos et al., 2018b; García-Ramos, Suzovic & Pérez-Castilla, 2019; Loturco et al., 2017; Sánchez-Medina et al., 2014; Torrejon et al., 2018). The popularity of the bench press is justified because it is one of the most effective exercises for improving upper-body strength and power (Castillo et al., 2012) and it is also commonly used by older individuals during their resistance training programs (Fatouros et al., 2005; Bavaresco Gambassi et al., 2019). Strong relationships between movement velocity and the %1RM have been reported for the bench press exercise irrespective of whether the exercise was performed in a Smith machine (*r*^2^ = 0.97) or with a free-weight barbell (*r*^2^ = 0.96) (Loturco et al., 2017). Similarly, a comparable strength of the load-velocity relationship in the bench press was observed for men (*r*^2^ = 0.95–0.97) and women (*r*^2^ = 0.94) (García-Ramos, Suzovic & Pérez-Castilla, 2019; Torrejon et al., 2018). However, although the strength of the load-velocity relationship did not significantly differ between the different studies (*r*^2^ ranged from 0.94 to 0.97), the results of these studies suggested that the respective equations provided for each exercise mode (Smith machine and free-weight) and sex (men and women) should be recommended to improve the precision in the estimation of the %1RM. It should be noted that the strength of the load-velocity relationship observed in the present study (*r*^2^ = 0.83) was considerably lower when compared to previous studies carried out with young and healthy individuals (*r*^2^ ≥ 0.94) (García-Ramos et al., 2018b; García-Ramos, Suzovic & Pérez-Castilla, 2019; Loturco et al., 2017; Sánchez-Medina et al., 2014; Torrejon et al., 2018). It is plausible that a higher number of familiarization sessions would have been necessary to increase the accuracy of the load-velocity relationship (Ploutz-Snyder & Giamis, 2001). More importantly the MV associated with submaximal loads (i.e., %1RM) in the present study tended to be lower compared to what has been reported in previous studies for young healthy subjects. For example, the MV associated with the 50%1RM in this study was 0.48 m · s^−1^, while a MV of 0.48 m · s^−1^ represented a load which would be approximately 78% of the 1RM in the study of Sánchez-Medina et al. (2014). These results reinforce the idea that velocity-based training should be individualized and that the use of general equations to estimate the %1RM from movement velocity may present limited value when they are extrapolated to other populations.

To our knowledge, the load-velocity relationship during the leg press exercise has only been examined by Conceição et al. (2016). These authors revealed a very strong relationship between movement velocity and the %1RM in a group of male track and field athletes (*r*^2^ = 0.96). A weaker association was observed in our study (*r*^2^ = 0.91). However, what is of even more importance is the meaningful differences in the velocity associated with each %1RM between the older women evaluated in the present study and the male athletes assessed by Conceição et al. (2016). In line with what has been observed for the bench press, the velocities associated with the same submaximal loads were also lower for older women. For example, the MV associated with the 50%1RM in our study was 0.49 m · s^−1^, while a MV of 0.49 m · s^−1^ represented approximately the 82%1RM in the study of Conceição et al. (2016). These results highlight that the general equations obtained with young and healthy individuals to estimate the %1RM from movement velocity should not be extrapolated to older women. However, it seems that the MV of the 1RM is comparable for older women (0.175 ± 0.017 m · s^−1^ in bench press and 0.210 ± 0.019 m · s^−1^ in leg press) and young healthy individuals (Conceição et al., 2016; García-Ramos et al., 2018b, 2019; Loturco et al., 2017; Sánchez-Medina et al., 2014; Torrejon et al., 2018). The lower velocities against submaximal loads, but not for the 1RM, observed in the present study for older women is in line with previous findings suggesting that aging produces a higher impairment in power production than in maximal strength capacity (Fernandes, Lamb & Twist, 2018).

This is the first study that has directly compared the load-velocity relationship between the bench press and leg press exercises. No significant differences were observed for the MV attained against light-medium relative loads (≤70%1RM), while the MV associated with heavy loads (≥80%1RM) was significantly higher for the leg press. These results partially contradict the previous literature that has reported higher velocities for the leg press compared to the bench press for all loads. For example, Conceição et al. (2016) reported a mean propulsive velocity of 1.40 m · s^−1^ for the 30%1RM and 0.19 m · s^−1^ for the 100%1RM in the leg press, while Sánchez-Medina et al. (2014) reported a mean propulsive velocity of 1.29 m · s^−1^ for the 30%1RM and 0.17 m · s^−1^ for the 100%1RM in the bench press. The cause of these discrepancies could be that older women have a higher deficit in force production against submaximal loads for the leg press than for the bench press. Finally, the high between-subject variability (overall CV = 15.5% and 8.6% for the bench press and leg press, respectively) further highlight the existence of individual load-velocity profiles (García-Ramos et al., 2018b; Helms et al., 2017; Pestaña-Melero et al., 2018).

A number of limitations and directions for future research should be addressed. Firstly, only women were assessed in the present study and, therefore, it remains to be elucidated whether the results of this study are applicable to older men. Secondly, future studies should evaluate both young and old adults in the same experiment because the differences observed in the present study could be partially mediated by the different testing procedures used. It is plausible that the weaker load-velocity relationship observed in the present study for the bench press could be partially explained because the bench press was performed with free-weights and not in a Smith machine as in the majority of previous studies. Finally, the inclusion of a holdout sample would have been desirable to evaluate the accuracy of the equations proposed in the present study for other subjects. However, we want to highlight that although the present findings do not necessarily support the use of these equations for the whole population of older women, it does reveal that the general load-velocity relationships previously proposed for young individuals should not be extrapolated to older adults. It is plausible that the modeling of the individual load-velocity relationship could also provide more valuable information for older people.

## Conclusions

The results of this study suggest that movement velocity can provide useful information to prescribe the relative load (%1RM) during resistance training sessions in older adults. However, it is important to note that (I) the precision of the load-velocity relationship seems to be weaker compared to what has been reported for young individuals, and (II) the velocities associated with each %1RM described for young individuals cannot be extrapolated to older women. Older women seem to present lower velocities for submaximal loads that young healthy adults. Future studies should be conducted to further explore the applications of velocity-based training in older adults.

## Supplemental Information

10.7717/peerj.7533/supp-1 Supplemental Information 1 Final results in leg press and bench press.

## Additional Information and Declarations

### Data Availability

The following information was supplied regarding data availability: The raw measurements are available as a Supplemental File.
